# The young, not-so-young, and the 2007 *Retrovirology *Prize: call for nominations

**DOI:** 10.1186/1742-4690-4-64

**Published:** 2007-09-17

**Authors:** Kuan-Teh Jeang

**Affiliations:** 1 National Institutes of Health, Bethesda, MD, USA

## Abstract

Recent findings suggest an aging scientific work force and an almost static publishing productivity in the United States. The *Retrovirology *Prize seeks to recognize and encourage the work of a mid-career retrovirologist between the ages of 45 and 60. The 2006 *Retrovirology *Prize was awarded to Dr. Joseph G. Sodroski.

## Ages 38 and 42

Recent US budgetary constraints on scientific research have prompted a discussion on the aging of academic faculty members and how this potentially impacts the career development of younger colleagues [1]. Two age-milestones hold significance for today's American scientists. 38 is now the average age that a US doctorate receives his/her first "real" job (i.e. a tenure track position); and, he/she will wait 5 additional years to secure his/her first R01 grant – the US National Institutes of Health's workhorse mechanism for supporting independent research. Indeed, since the 1970s, the age that a US investigator wins his/her first R01 has risen steadily from 34 to 42. The scenario for the "young" is no more optimistic in other countries. The average age that today's German scientist receives his/her first independent grant is 41; and in 2007, scientists over the age of 50 compose 50% of successful applicants for Australian Research Council grants, while only 6% of successful scientists are in the 30–34 age range.

Perhaps more daunting is the finding that while three decades ago, nearly 45% of all US biological doctorates held tenure or tenure-track jobs, today that proportion has fallen to fewer than 30% [2]. Also troubling is a recent report from the US National Science Foundation (NSF) which revealed that American scientists and engineers have not increased recently their rate (an average of <1% increase per year from 1992 to 2003) of scientific publishing in peer-reviewed journals while the commensurate metrics for East Asian countries such as China, Singapore, South Korea and Taiwan have grown at an annual rate approximating 16 percent [3]. There is no evidence that the plateau in American publishing is related to an aging scientific workforce. Nonetheless, common sense intuition suggests that more attention on constructive nurturing, encouragement, and support of young and mid-career scientists might serve to alleviate a productivity bottleneck.

## Ages 45 to 60 and the *Retrovirology *Prize

**Figure 1:**
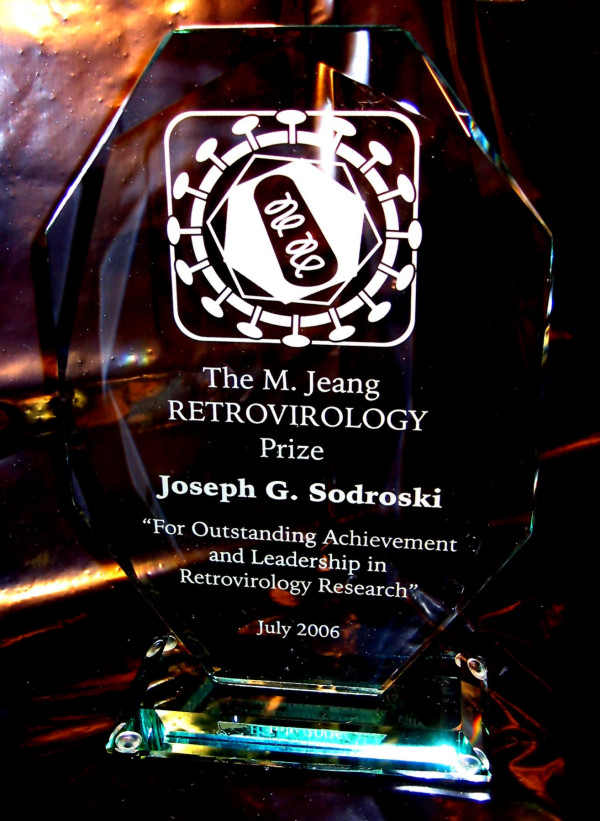
A photograph of the crystal trophy presented to Dr. Joseph G. Sodroski, winner of the 2006 M. Jeang *Retrovirology *Prize.

Annually, the *Retrovirology *Prize recognizes the outstanding efforts of a "young, mid-career" retrovirologist between the ages of 45 to 60 [4]. The Prize consists of an attractive crystal trophy (Figure 1), a $3,000 cash award, and a profile article of the awardee published in *Retrovirology*. The *Retrovirology *Prize is supported in part through a donation from the Ming K. Jeang Foundation, an educational foundation based in Houston, Texas, USA. Accordingly, the Prize is named the M. Jeang *Retrovirology *Prize.

In 2005, Dr. Stephen P. Goff of Columbia University was our winner [5]. In 2006, Dr. Joseph G. Sodroski of the Dana Farber Cancer Institute was the awardee [6].

## Call for nominations and the selection process

As stated previously [4], the Prize alternates yearly between recognizing a non-HIV retrovirologist (2007 and odd years) and an HIV retrovirologist (2006 and even years). There can be some discretion on this guideline exercised from time-to-time by the selection committee. Any individual can initiate a nomination of others or self-nominate. A nomination includes a statement (1000 words or less) of the nominee's significant contributions to retrovirus research, a curriculum vitae of the nominee, and a statement by the nominator that the nominee has agreed to be nominated. The selection committee consists of the Editors of *Retrovirology *(currently, M. Benkirane, B. Berkhout, M. Fujii, K.T. Jeang, M. Lairmore, A. Lever, and M. Wainberg). All nominations submitted to the selection committee must be communicated through an Editorial Board member of *Retrovirology*. Hence, an individual who is not an Editorial board member but who wishes to make a nomination should seek out a *Retrovirology *Editorial board member to communicate his/her information to the selection committee. A list of current Editorial Board members can be found at the *Retrovirology *website [7]. Within stipulated age limits, all *Retrovirology *Editors and Editorial Board members are eligible to be nominated with the exception of the Editor-in-Chief who will administer the final selection.

For 2007, nominations will begin September 15^th ^and will close October 30^th^. I urge all members of the retrovirology community to participate in this process for recognizing a deserving colleague.
